# Quantitative Assessment of Changes in Hemodynamics After Obliteration of Large Intracranial Carotid Aneurysms Using Computational Fluid Dynamics

**DOI:** 10.3389/fneur.2021.632066

**Published:** 2021-04-29

**Authors:** Yongsheng Liu, Guinan Jiang, Feng Wang, Xiangbo An

**Affiliations:** Department of Interventional Neuroradiology, First Affiliated Hospital of Dalian Medical University, Dalian, China

**Keywords:** carotid artery, hemodynamics, large intracranial aneurysm, computational fluid dynamics, geometry

## Abstract

**Background:** It was speculated that the alteration of the geometry of the artery might lead to hemodynamic changes of distal arteries. This study was to investigate the hemodynamic changes of distal arterial trees, and to identify the factors accounting for hyperperfusion after the obliteration of large intracranial aneurysms.

**Methods:** We retrospectively reviewed data of 12 patients with intracranial carotid aneurysms. Parametric models with intracranial carotid aneurysm were created. Patient-specific geometries were generated by three-dimensional rotational angiography. To mimic the arterial geometries after complete obliteration of the aneurysms, the aneurysms were virtually removed. The Navier–Stokes equations were solved using ANSYS CFX 14. The average wall shear stress, pressure and flow velocity were measured.

**Results:** Pressure ratio values were significantly higher in A1 segments, M1 segments, and M2 + M3 segments after obliteration of the aneurysms (*p* = 0.048 in A1 segments, *p* = 0.017 in M1 segments, *p* = 0.001 in M2 + M3 segments). Velocity ratio values were significantly higher in M1 segments and M2 + M3 segments after obliteration of the aneurysms (*p* = 0.047 in M1 segments, *p* = 0.046 in M2 + M3 segments). The percentage of pressure ratio increase after obliteration of aneurysms was significantly correlated with aneurysmal angle (*r* = 0.739, *p* = 0.006 for M2 + M3).

**Conclusions:** The pressure and flow velocity of distal arterial trees became higher after obliteration of aneurysms. The angle between the aneurysm and the parent artery was the factor accounting for pressure increase after treatment.

## Introduction

Intracranial aneurysm is a common disease in the general population (1). Cerebral hyperperfusion syndrome (HPS) and remote intracerebral hemorrhage (ICH) after treatment of the large intracranial aneurysm have been noted (2–5). The mechanism remains unknown, but it was speculated that the alteration of the geometry of the artery might lead to hemodynamic changes of distal arterial trees, which may contribute to cerebral HPS and remote ICH; nonetheless, this speculation has not been well studied (2, 4, 6–8).

With the development of computational fluid dynamics (CFD) and three-dimensional imaging technology, patient-specific hemodynamic analysis has become feasible. However, quantitative study of the hemodynamic changes that occur in the region distal to the aneurysms after the obliteration of large intracranial aneurysms is relatively rare (6).

The aim of our study was to investigate the hemodynamic changes of distal arterial trees after the obliteration of large intracranial carotid aneurysms and to identify the factors accounting for hyperperfusion after the obliteration of large intracranial carotid aneurysms.

## Methods

### Patient Selection

We retrospectively reviewed data of 12 patients with large intracranial aneurysms of internal carotid artery (ICA) between August 2018 and August 2019 in our institution.

Inclusion criteria were unilateral intracranial aneurysms of ICA and the maximum diameter of aneurysm ≥10 mm. Exclusion criteria were stenosis of ICA and multiple cerebral aneurysms.

### Modeling of the Aneurysms and Hemodynamic Parameter Calculations

The angles between parent artery and the aneurysm were measured.

**Figure 1:**
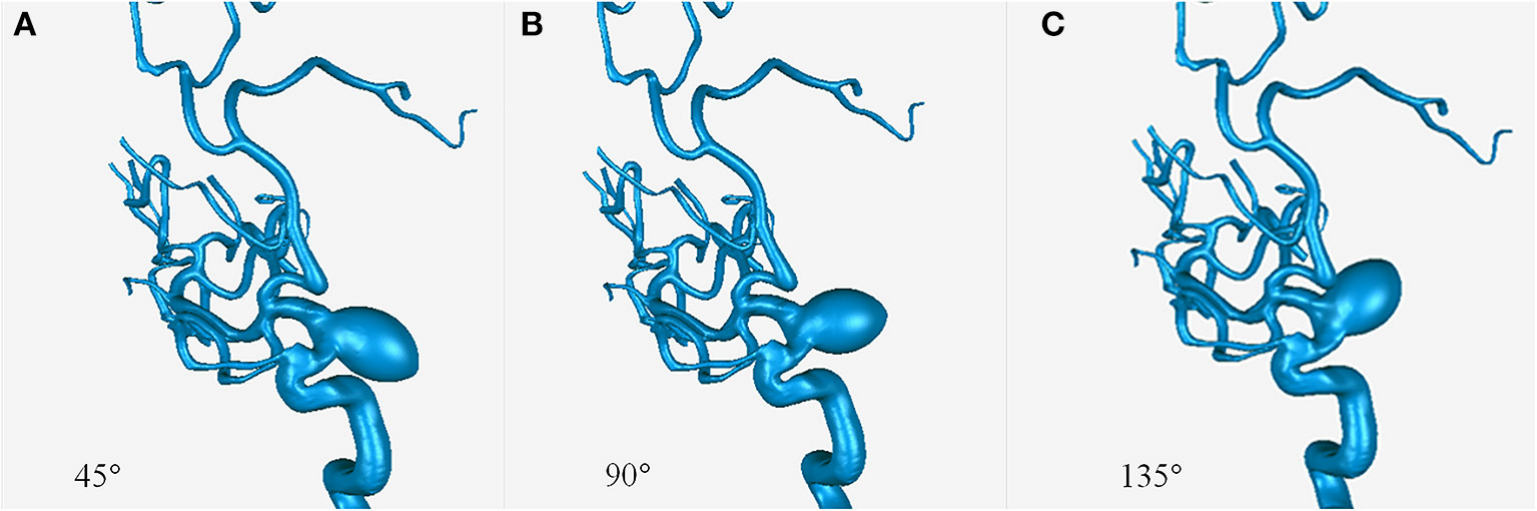
Models with aneurysmal angles of 45° **(A)**, 90° **(B)**, and 135° **(C)**.

Parametric models with an aneurysm of ICA (15–10–10 mm) derived from patient 11 were created using SolidWorks software (SolidWorks Co, Concord, MA, USA). Three models were created with aneurysmal angles of 45°, 90°, and 135° (Figure 1).

Patient-specific geometries were generated by three-dimensional rotational angiography. The surface images were reconstructed using Mimics software (Materialize Co., Leuven, Belgium). To mimic the arterial geometries after complete obliteration of the aneurysms, the aneurysms were virtually removed.

After segmenting and surface smoothing by Geomagic Studio 9.0 software (Geomagic USA), the geometries in stereolithography (STL) format were then exported to ICEM CFD 14.0 (ANSYS, Inc., Canonsburg, PA, USA) for meshing. The vessels were assumed to be rigid with no-slip boundary conditions.

The blood was assumed as incompressible fluid with a density of 1,025 kg/m^3^ and a viscosity of 0.0035 Pa s. The walls of the patient geometry were assumed as rigid (9). The inlet boundary condition used mass-flow boundary condition (245 ml/min) (10), whereas the outlet boundary condition used pressure outlet with zero pressure. The Navier–Stokes equations were solved using ANSYS CFX 14.0 (ANSYS, Inc., Canonsburg, PA, USA).

The beginning part of the cavernous segment of ICA was defined as the origin plane.

The average wall shear stress (WSS), pressure, and flow velocity were measured. We therefore normalized them to achieve the relative indices such as WSS ratio, velocity ratio and pressure ratio, divided by the values of the corresponding origin plane (11).

### Statistical Analysis

Comparisons of hemodynamic parameters of the middle cerebral arteries (MCAs) and anterior cerebral arteries (ACAs) between the pre-obliteration group and post-obliteration group were performed.

SPSS 19.0 software (SPSS Inc., Chicago, IL, USA) was used for statistical analyses. Statistical significance was assessed by the application of paired *t*-tests comparing the hemodynamics between the pre-obliteration group and post-obliteration group. The association between the percentage of pressure ratio increase after obliteration of aneurysms and aneurysmal angles was quantified using Pearson's correlation coefficients. All tests used a significance level of *p* < 0.05.

## Results

### Patient Characteristics

The study population comprised 12 patients, including nine females (75%) and three males (25%). The patient characteristics and the aneurysm features are summarized in Table 1.

**Table 1:** Characteristics of the study population.

**Subject**	**Sex**	**Age (y)**	**Smoking**	**Diabetes mellitus**	**Hypertension**	**Location**	**Side**	**Size (mm)**	**Aneurysmal angle (^**°**^)**
1	M	53	Yes	Yes	Yes	Cavernous	R	14–13–10	41
2	F	73	No	No	Yes	Posterior communicating	L	12–8–7	79
3	F	61	No	No	Yes	Ophthalmic	L	11–6–6	71
4	F	40	No	No	No	Posterior communicating	L	10–6–5	82
5	F	53	No	No	No	Posterior communicating	R	23–20–16	95
6	F	57	No	No	No	Ophthalmic	L	20–20–17	96
7	F	52	No	No	No	Posterior communicating	L	11–7–7	135
8	F	65	No	No	Yes	Posterior communicating	L	20–20–17	51
9	M	54	Yes	No	No	Posterior communicating	R	21–14–10	65
10	F	72	No	No	Yes	Paraclinoid	R	13–10–7	105
11	M	62	No	No	Yes	Posterior communicating	R	11–8–8	96
12	F	66	No	No	Yes	Posterior communicating	L	13–11–9	80

*L, left; R, right*.

### Hemodynamics

**Figure 2:**
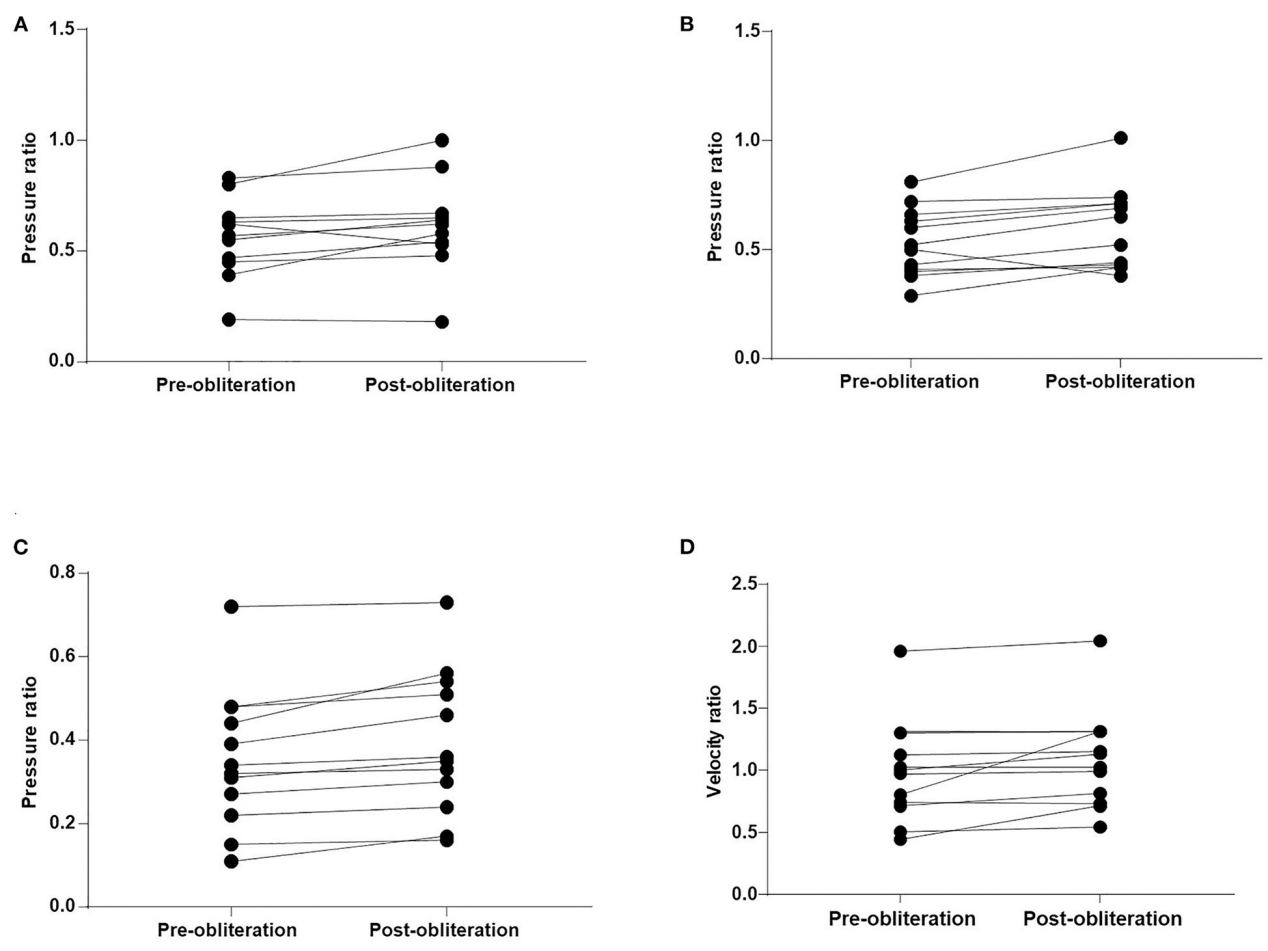
Comparison of the pressure ratio and velocity ratio after obliteration of aneurysms. **(A)** A1 segments. **(B)** M1 segments. **(C)** M2 + M3 segments. **(D)** M2 + M3 segments.

**Figure 3:**
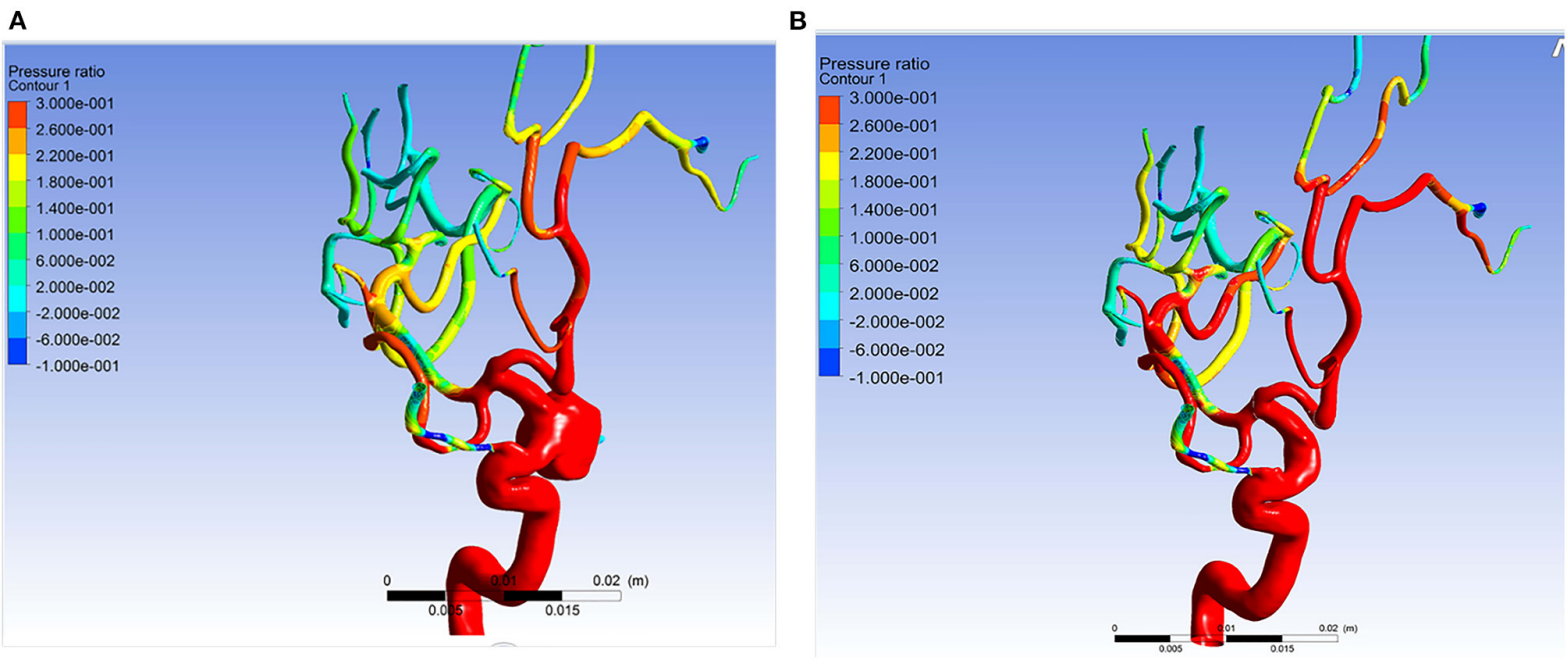
Example of simulation results of patient 11. The pressure ratio in the arteries distal to aneurysm became higher after the aneurysm was obliterated. **(A)** The pressure ratio before obliteration of the aneurysm. **(B)** The pressure ratio after obliteration of the aneurysm.

The WSS ratio, velocity ratio, and pressure ratio values were analyzed (Table 2, Figures 2, 3). Statistical analysis demonstrated that pressure ratio values were significantly higher in A1 segments, M1 segments, and M2 + M3 segments after obliteration of the aneurysms (*p* = 0.048 in A1 segments, *p* = 0.017 in M1 segments, *p* = 0.001 in M2 + M3 segments). Velocity ratio values were significantly higher in M1 segments and M2 + M3 segments after obliteration of the aneurysms (*p* = 0.047 in M1 segments, *p* = 0.046 in M2 + M3 segments). The WSS ratio values were similar in MCAs and ACAs for both groups (Table 2).

**Table 2:** Hemodynamic changes in the distal arteries after obliteration of aneurysms.

	**Mean** **±** **SD**		***P*-value**
	**Pre-obliteration**	**Post-obliteration**	
**A1 segments**			
WSS ratio	3.33 ± 3.84	3.25 ± 3.11	0.732
Pressure ratio	0.56 ± 0.18	0.62 ± 0.21	0.048
Velocity ratio	1.35 ± 0.64	1.38 ± 0.57	0.368
**A2 segments**			
WSS ratio	2.11 ± 2.01	2.11 ± 1.77	0.952
Pressure ratio	0.44 ± 0.14	0.46 ± 0.15	0.129
Velocity ratio	1.10 ± 0.45	1.16 ± 0.41	0.190
**A3 segments**			
WSS ratio	2.21 ± 2.88	2.16 ± 2.48	0.730
Pressure ratio	0.28 ± 0.16	0.27 ± 0.10	0.746
Velocity ratio	1.05 ± 0.61	1.13 ± 0.16	0.246
**M1 segments**			
WSS ratio	4.48 ± 5.49	4.02 ± 3.33	0.519
Pressure ratio	0.53 ± 0.16	0.59 ± 0.19	0.017
Velocity ratio	1.61 ± 0.76	1.69 ± 0.72	0.047
**M2** **+** **M3 segments**			
WSS ratio	1.94 ± 1.84	2.04 ± 1.78	0.076
Pressure ratio	0.35 ± 0.17	0.39 ± 0.17	0.001
Velocity ratio	0.99 ± 0.41	1.09 ± 0.40	0.046

**Figure 4:**
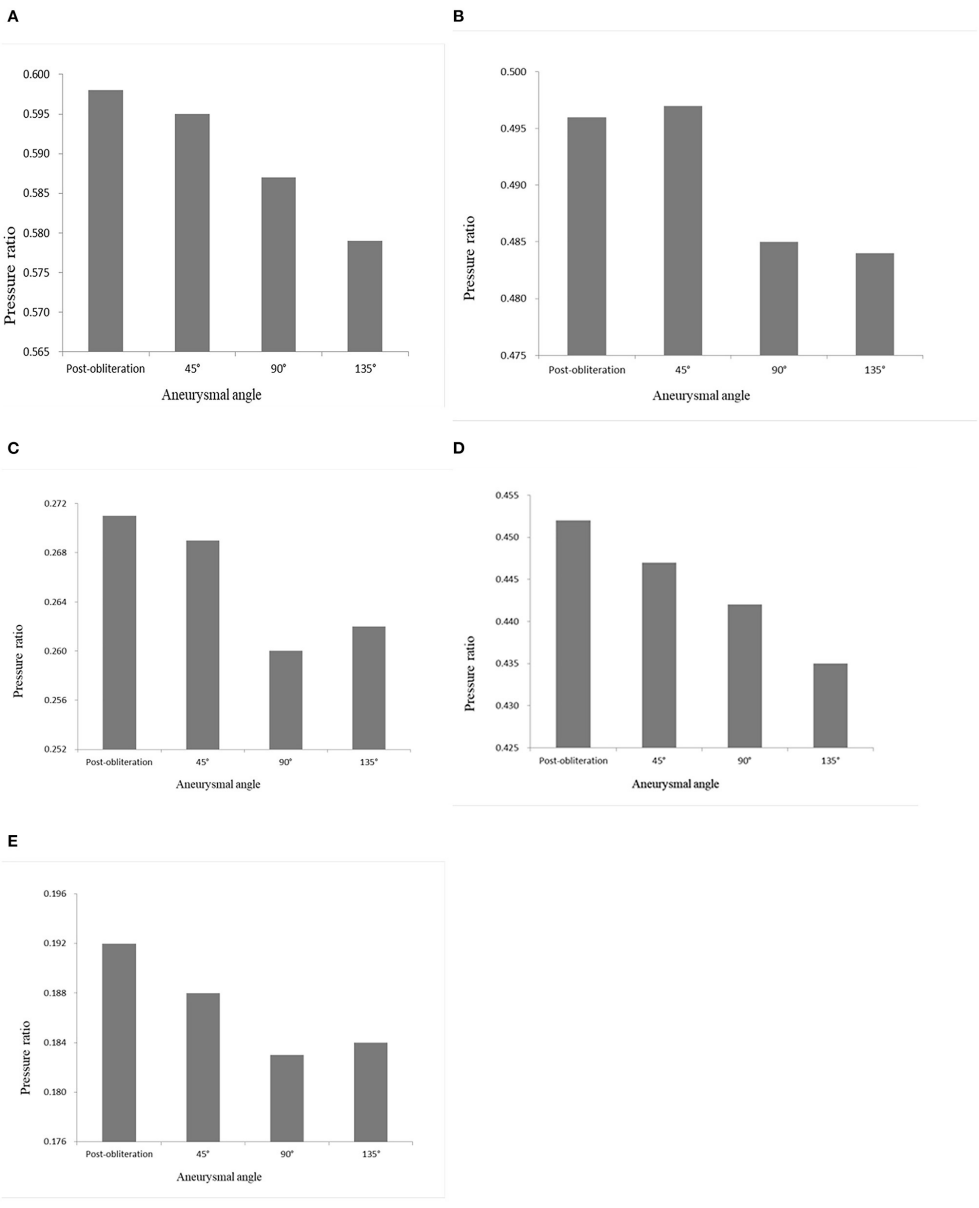
Pressure ratio of the models with aneurysmal angles of 45°, 90°, 135°, and the no-aneurysm model. **(A)** A1 segment. **(B)** A2 segment. **(C)** A3 segment. **(D)** M1 segment. **(E)** M2 + M3 segments.

CFD study of the parametric models showed that an increasing aneurysmal angle yielded a lower pressure ratio of ACAs and MCAs (Figure 4). Therefore, the aneurysmal angle might influence the pressure change of distal arterial trees after obliteration of aneurysms.

**Figure 5:**
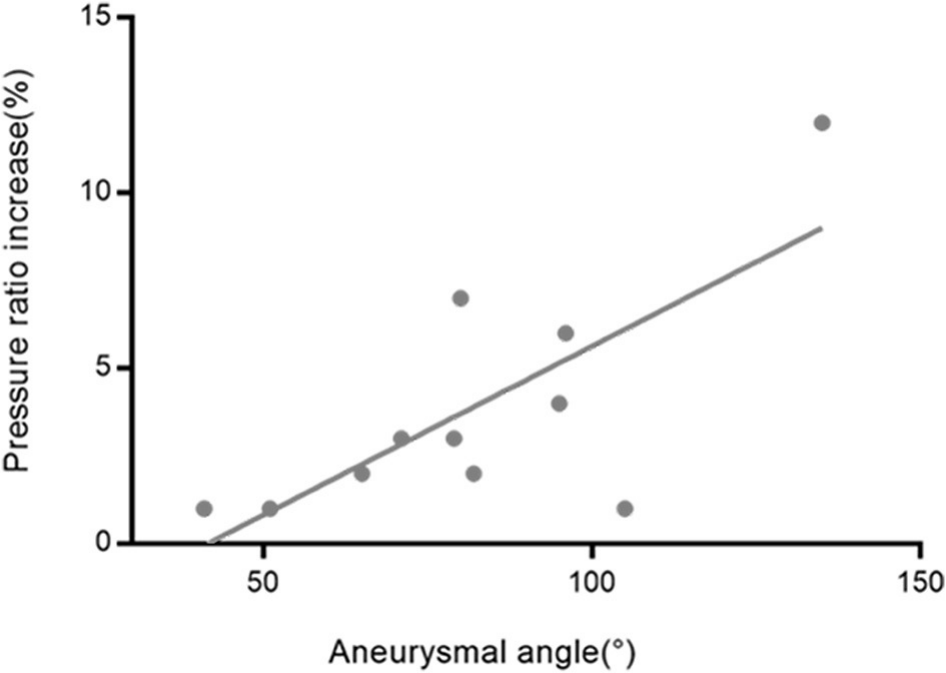
The relationship between the aneurysmal angles and the pressure ratio increase rates in M2 + M3 segments after obliteration of the aneurysms. The correlation coefficient was *r* = 0.739.

The percentage of the pressure ratio increase after obliteration of aneurysms was significantly correlated with aneurysm angle (*r* = 0.739, *p* = 0.006 for M2 + M3) (Figure 5). The percentage of the pressure ratio increase after obliteration of aneurysms was not significantly correlated with aneurysm volume (Pearson *r* = −0.018, *p* = 0.958 for A1; *r* = −0.035, *p* = 0.914 for M1; *r* = −0.139, *p* = 0.667 for M2 + M3). The percentage of the pressure ratio increase in A1 and M1 segments after obliteration of aneurysms was not significantly correlated with aneurysmal angle (*r* = −0.113, *p* = 0.741 for A1; *r* = −0.022, *p* = 0.945 for M1). The percentage of the pressure velocity increase after obliteration of the aneurysms was not significantly correlated with the aneurysm angle (*r* = −0.549, *p* = 0.065 for M2 + M3).

## Discussion

Cerebral HPS and remote ICH are unpredictable and potentially severe complications after treatment of the large intracranial aneurysm. Among all the possible etiologies (2, 12–14), hemodynamic changes have been proposed as a possible candidate, but no mechanism has been well-studied (15).

The relationship of geometry and hemodynamics is mutually causal. The hemodynamics of arteries distal to the aneurysms may be changed after obliteration of aneurysms. Many studies have concentrated on the hemodynamic changes within the aneurysms after treatment of intracranial aneurysms. However, hemodynamic changes within distal arterial trees after aneurysm treatment are much less understood.

The incidence of remote ICH appears to be higher for large aneurysms than for small aneurysms (8, 14–16). Therefore, we hypothesized that pressure and velocity of distal arteries might become higher after obliteration of large aneurysms.

After obliteration of aneurysms, blood flow through the vessels distal to the aneurysms may suddenly increase. Increased flow rate and pressure distal to the aneurysms after clipping or endovascular treatment have been demonstrated in several studies. Brunozzi et al. reported that the ratio of ipsilateral MCA to systemic systolic and mean blood pressure increased after flow diverter device deployment (7). In our study, statistical analysis demonstrated that pressure ratio values became higher in MCAs and A1 segments after obliteration of aneurysms. Compared to the ACAs, MCAs had a higher pressure increase, indicating the higher risk of HPS in the areas supplied by MCAs.

There are few findings regarding risk factors of the appearance of hyperperfusion after obliteration of aneurysms (7). Brunozzi et al. reported that the hemodynamic changes in the arteries distal to the aneurysms after flow diverter device deployment were independent from aneurysm size (7). Our CFD study of the models suggested that a large aneurysm can induce pressure loss, resulting in hyperperfusion after obliteration of aneurysm. The aneurysmal angle was the factor accounting for pressure loss.

According to the results of our study, the percentage of the pressure ratio increase after obliteration of aneurysms was not correlated with aneurysm volume. Our study suggested that the angle between the aneurysm and the parent artery was the factor accounting for the pressure increase after obliteration of aneurysms. It was only a preliminary finding based on the results of our study. Further studies are required to identify which patients are at a higher risk of hyperperfusion after obliteration of aneurysms.

Brunozzi et al. demonstrated that the mean flow velocity of MCA increased especially in patients with delayed ipsilateral ICH after flow diverter device deployment (6). Chiu et al. reported a case of increasing cerebral blood flow and cerebral blood volume distal to the aneurysm after flow diverter treatment (3). In our study, velocity ratio values became higher in M2 + M3 segments after obliteration of aneurysms.

Prevention of HPS is critical. Several investigators have found that careful monitoring and comprehensive management of blood pressure can lower the incidence of HPS after carotid artery stenting (17, 18). Blood pressure reduction may lower the pressure of cerebral arteries and reduce the risk of HPS after obliteration of aneurysms.

WSS can be viewed as the frictional force applied against the vascular wall by the movement of blood. Study of the WSS of the arteries distal to the aneurysms after the obliteration of large intracranial aneurysms is sparse. In a study by Shakur et al., the WSS values were higher in the ipsilateral MCA among patients with hemorrhage after flow diverter device placement (19). Our study demonstrated that the WSS ratio values were similar in MCAs and ACAs for both groups.

Limitations to this study include its retrospective nature, a small sample size, and single institution design. Further study with a larger number of patients would be necessary to validate our findings. Patient-specific flow-boundary information was unavailable, which might affect the results. Virtual aneurysm removal might underestimate or overestimate the size of the healthy lumen. This study only involved large intracranial aneurysms of ICAs, which limited the generalization of the study results.

## Conclusion

Pressure ratio values became higher in MCAs and A1 segments after obliteration of large intracranial carotid aneurysms. The angle between the aneurysm and the parent artery was the factor accounting for the pressure increase after treatment. Velocity ratio values became higher in M2 + M3 segments after obliteration of aneurysms.

## Data Availability Statement

The raw data supporting the conclusions of this article will be made available by the authors, without undue reservation.

## Ethics Statement

The studies involving human participants were reviewed and approved by Ethics Committee of First Affiliatted Hospital of Dalian Medical University. Written informed consent for participation was not required for this study in accordance with the national legislation and the institutional requirements.

## Author Contributions

YL and GJ carried out the simulation study and drafted the manuscript. GJ and XA performed the data collection and data analysis. YL and FW participated in the design of this study. XA helped to check the manuscript. All authors contributed to the article and approved the submitted version.

## Conflict of Interest

The authors declare that the research was conducted in the absence of any commercial or financial relationships that could be construed as a potential conflict of interest.
